# The Association of Myopia Progression with Changes in Retinal Thickness among Primary School Students with Myopia

**DOI:** 10.1155/2024/1055700

**Published:** 2024-08-06

**Authors:** Jing Shang Zhang, Jing Li, Jin Da Wang, Ying Xiong, Kai Cao, Meng Li, Kai Jie Wang, Ying Yan Mao, Jian Ying Liu, Xiu-Hua Wan

**Affiliations:** Beijing Tongren Eye Center Beijing Tongren Hospital of Capital Medical University Beijing Key Laboratory of Ophthalmology and Visual Sciences, Beijing 100005, China

## Abstract

**Purpose:**

To observe the relationship between myopia progression and changes in retinal thickness during one year of follow-up among primary school children.

**Methods:**

The study included 1161 eyes of 708 myopic children, with 616 (53.06%) right eyes and 545 (46.94%) left eyes. The participants underwent a comprehensive ophthalmic examination, including visual acuity, axial length (AL), autorefraction, and optical coherence tomography (OCT) examination in 2016 and in 2017. An analysis was conducted on the differences in retinal thickness between different genders and between high myopia and nonhigh myopia. Furthermore, the study delved into the correlation between the progression of myopia and the changes of retinal thickness.

**Results:**

The average diopter was −1.83 ± 1.29D, average AL was 23.78 ± 0.94 mm, and average foveal thickness was 228.02 ± 23.00 *μ*m. For the inner retina, the median value [the lower quartile value, the upper quartile value] of the foveal thickness was thicker in the high myopia group than the nonhigh myopia group (67 [64; 74] *μ*m vs. 63 [56; 70] *μ*m), while the parafoveal region and perifoveal region were thinner in the high myopia group than the nonhigh myopia group (106 [100; 123] *μ*m vs. 124 [117; 130] *μ*m; 95.0 [93; 102] *μ*m vs. 104 [100; 108] *μ*m). Among all the children with myopia, 67.53% (784/1161) of them have a diopter progression within one year. The AL progression was 95.43% (1108/1161). The retinal thickness of all children has slightly increased in various regions. As the AL of the eye increased and the diopter decreased, the progression degree of inner retinal thickness and full retinal thickness (exclusive of full fovea) decreased.

**Conclusion:**

For the school-age myopic children, the inner foveal retinal thickness were thicker in highly myopic students than in the nonhighly myopic students, while the parafoveal and perifoveal retina were thinner in highly myopic students. The inner and full retinal thicknesses of male students were thicker than that of females. The progression of myopia mainly affected the changes of the inner retinal thickness in the one-year follow-up.

## 1. Introduction

Myopia is becoming increasingly common around the world. Myopia affects 30% of the world's population today, and it is anticipated to affect nearly half of the population by 2050 [1]. The excessive increase of the myopic diopter and AL may lead to the thinning of the retina [2, 3]. Studies on the thinning of the central regions of the retina in patients with high or pathological myopia have been carried out [4, 5]. Meanwhile, the study on the changes of thickness in different retinal regions (fovea, parafovea, and perifovea) and layers (full, inner, and outer retinas) in nonmyopic and myopic participants was also conducted [6]. However, the change of thickness in different regions of retina between high myopia and nonhigh myopia has not been clearly studied.

With the accelerated increase in the incidence and progression of myopia in school-age children, researchers are putting more focus on retinal thickness in children with myopia. A study on 11-year-old myopic children revealed thickened macular fovea and thinned parafovea, indicating changes in retinal thickness as an early indicator of myopia development. Moreover, the consistent thinning of the parafoveal retina in the inferior region suggests the potential of monitoring regional retinal changes to track myopia progression at an early stage [7]. To gain a deeper understanding of the anatomical and morphological alterations in the retinal layer among children with different refractive conditions, a cross-sectional study was conducted to examine retinal thickness [8]. Read et al. further explored macular thickness and the thickness of each retinal layer, discovering that in myopic children aged 10–15 years, the outer and inner layers surrounding the fovea tend to become thinner [9]. However, the changes in retinal thickness in children with myopia during follow-up may require further observation and analysis.

Therefore, this study compared and analyzed the distribution of retinal thickness in different regions among school-age children with high myopia and nonhigh myopia. At the same time, it conducted a one-year follow-up on these students with myopia, observing and analyzing the association between the progression of myopia and changes in retinal thickness in different regions.

## 2. Methods

### 2.1. Participants

The current school-based, prospective investigation invited children from six primary schools in Sanhe city of Hebei province in Northern China to participate in the Sanhe Cohort Study of the Risk Factors for Myopia (SCSRFM) [10]. At the baseline, children with eye diseases or undergoing myopia control interventions, such as low concentration atropine and orthokeratology, were excluded. The study was approved by the Institutional Review Board of Beijing Tongren Hospital (QN20150228), Capital Medical University, and the protocol adhered to the Declaration of Helsinki.

The study included 1161 eyes of 708 myopic children (the diopter ≤0.50*D*), with 616 (53.06%) right eyes and 545 (46.94%) left eyes. The participants underwent a comprehensive ophthalmic examination in 2016 and in 2017. There were 346 males (48.87%) and 362 females (51.13%), with an average age of 10.11 ± 0.56 (8–11) years in 2016. Data on diopter, AL, and optical coherence tomography (OCT) were recorded in 2016 and in 2017 [11]. High myopia was defined as a spherical equivalent (SE) refractive error ≤−6.00 diopters (*D*) or the AL ≥26 mm.

### 2.2. Ocular Examination

The participants underwent a comprehensive ophthalmic examination, including visual acuity measurement, autorefraction, and posterior segment examination. The Lenstar LS 900 (Haag-Streit, Koeniz, Switzerland) was used to measure AL. Ametropia was confirmed with subjective refraction, and the automatic refractometer RK-3000 (Topcon, Tokyo, Japan) without cycloplegia was used for the objective refraction to generate the reference value. The retinal thickness of each region was collected using the RTVue spectral domain OCT (Optovue Inc., remont, CA, USA). All examinations were performed by trained ophthalmologists and optometrists.

### 2.3. OCT Measurements

**Figure 1:**
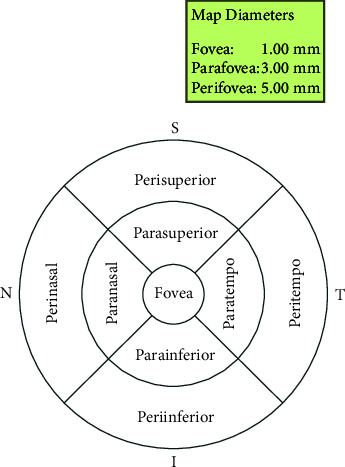
The map of the different retinal region.

**Figure 2:**
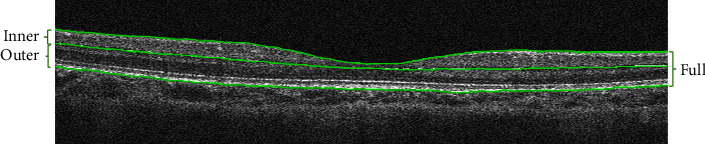
The boundary retinal thickness segmented by OCT software.

Both eyes of all students underwent OCT examination without cycloplegia. Only the OCT images with signal strength greater than 50% of maximal strength and without imaging artifacts or distortion were included. All scans examined the entire thickness of the retina. Using RTVue OCT instrument software, the whole retinal thickness was automatically measured in three concentric annular areas as follows: 1 mm centered on the fovea, the parafoveal region was 1∼3 mm diameter circle near the fovea, and the perifoveal region of 3∼5 mm diameter circle around the parafoveal region. The nine areas of OCT detection of retinal maps were shown by the standard early treatment of diabetic retinopathy (ETDRS) form, and the parafoveal region and perifoveal region were divided into tempo, nasal, superior, and inferior subregions (Figure 1) [6]. The analysis was conducted based on the boundary retinal thickness segmented by OCT software, namely, the full retinal thickness (from inner limiting membrane to outer retinal pigment epithelium), the thickness of the inner retina (from the inner limiting membrane to the outer boundary of the inner plexiform layer), and the thickness of the outer retina (from the outer boundary of the inner plexiform layer to the outer retinal pigment epithelium) (Figure 2).

### 2.4. Statistical Analysis

Normally distributed data were expressed as the mean value ± standard deviation, and the differences between males and female was analyzed using the *T*-test. As for the data of the high myopia group and the nonhigh myopia group were not normally distributed, the median value and interquartile range (the median value [the lower quartile value, the upper quartile value]) were used for statistical description, and the differences between the two groups were analyzed using the Wilcoxon rank-sum test. The correlation between the parameters was explored using Spearman's rank correlation coefficients. The value of progression mainly refers to the mean ± standard deviation of the difference between 2017 and 2016 for various parameters. *P* < 0.05 was considered to be statistically significant. All analyses were performed using SPSS software version 20.0 (SPSS, Inc., IBM, NY, USA).

## 3. Results

### 3.1. Thickness of Different Retinal Regions in Baseline

The average diopter was −1.83 ± 1.29D and the average AL was 23.78 ± 0.94 mm in all participants. The average foveal thickness was 228.02 ± 23.00 *μ*m, with an inner layer thickness of 63.79 ± 10.88 *μ*m and an outer layer thickness of 164.23 ± 14.13 *μ*m. The average parafoveal thickness was 310.35 ± 13.22 *μ*m, with an inner layer thickness of 122.02 ± 11.30 *μ*m and an outer layer thickness of 188.33 ± 10.31 *μ*m. The average perifoveal thickness was 279.26 ± 12.96 *μ*m, with an inner layer thickness of 103.80 ± 6.90 *μ*m and an outer layer thickness of 175.55 ± 9.18 *μ*m (See Table 1 for details).

### 3.2. Analysis of the Thickness of Different Retinal Regions in the High Myopia Group (≤−6.00D) and the Nonhigh Myopia Group (>−6.00D) at the Baseline

The participants were divided into the following two groups according to the diopter: the high myopia group (≤−6.00D) and the nonhigh myopia group (>−6.00D). The average diopter was 7.30 ± 0.92D in the high myopia group (14 eyes) and 1.81 ± 1.45D in the nonhigh myopia group (1147 eyes). For the inner retina, the foveal thickness was thicker in the high myopia group than the nonhigh myopia group (67 [64; 74] *μ*m vs. 63 [56; 70] *μ*m), while the parafoveal region and perifoveal region were thinner in the high myopia group than in the nonhigh myopia group (106 [100; 123] *μ*m vs. 124 [117; 130] *μ*m; 95.0 [93; 102] *μ*m vs. 104 [100; 108] *μ*m). The situation in each subregions was consistent and had statistical differences (all *P* < 0.05). For the outer retina, all regions were thicker (with statistical differences in the regions of the fovea, parafovea, parasuperior, parainferior, and perinasal) in the high myopia group than in the nonhigh myopia group. In terms of the full-thickness retina, the fovea was thicker in the high myopia group than in the nonhigh myopia group (248 [238; 251] *μ*m vs. 229 [214; 243] *μ*m), while the parafoveal region and perifoveal region had no statistical differences (see Table 2 for details).

### 3.3. Analysis of the Thickness of Different Retinal Regions in Subgroups with Different AL at the Baseline

The participants were divided into the AL ≥26 mm group and the AL <26mm group. The average AL was 26.69 ± 0.47 mm in the AL ≥26 mm group (15 eyes) and 23.79 ± 0.88 mm in the AL <26 mm group (1146 eyes). For the inner retina, the foveal thickness was thicker in the AL ≥26 mm group than that in the AL <26 mm group (70 [66; 74] *μ*m vs. 63 [56; 70] *μ*m), while the parafoveal region and perifoveal region were thinner in the AL ≥26 mm group than that in the AL <26 mm group (105 [102; 118] *μ*m vs. 124 [117; 130] *μ*m; 94 [91; 98] *μ*m vs. 104 [100; 108] *μ*m). The situation in each subregions was consistent and had statistical differences (all *P* < 0.05). For the outer retina, all regions were thicker (with statistical differences in the foveal and parafoveal region) in the AL ≥26 mm group than the AL <26 mm group. In terms of the full-thickness retina, the fovea was thicker in the AL ≥26 mm group than that in the AL <26 mm group (246 [238; 252] *μ*m vs. 229 [214; 243] *μ*m), while the parafoveal region and perifoveal region had no statistical differences (see Table 3 for details).

### 3.4. Analysis of the Thickness of Different Retinal Regions in Male and Female Participants at the Baseline

There were 346 males (564 eyes) and 362 females (597 eyes). The average diopter was −1.83 ± 1.30D in male participants and −1.92 ± 1.29D in female participants, without a statistical difference (*P* = 0.244). The average AL was 24.04 ± 0.91 mm in male participants and 23.63 ± 0.92 mm in female participants, with a statistical difference (*P* < 0.001). For the inner retina, outer retina, and full retina, the thickness of the foveal and parafoveal regions were thicker in males than females, and the situation in each subregions were consistent and had statistical differences (*P* < 0.05). For the perifoveal region, the thickness of inner retina was thinner in males than females (103.58 ± 6.85 *μ*m VS 104.01 ± 6.94 *μ*m), but it had no statistical differences (*P* = 0.294) (see Table 4 for details).

### 3.5. Progression of 1-Year Follow-Up

The mean of diopter progression was −0.27 ± 1.09D and the mean of AL progression was 0.33 ± 0.39 mm; the proportions of progression were 67.53% (784/1161) and 95.43% (1108/1161), respectively. The lower proportion of progression for diopter was possible that the subtle progression in the diopter could not be reflected due to the 0.25D deviation in autorefraction, which further led to the inconsistency. The retinal thickness of all children has slightly increased in various regions, with a greater increase in the foveal region than in the perifoveal region (5.50 ± 19.41 *μ*m vs. 1.69 ± 9.71 *μ*m) (see Table 5 for details).

### 3.6. Association of the Progression in AL and Diopter with the Progression in Retinal Thickness

Further analysis was carried out on the association of the progression in AL and diopter with the changes in the thickness of different retinal regions. As the AL of the eye increased and the diopter decreased, the progression degree of inner retinal thickness and full retinal thickness\(exclusive of full fovea) decreased, and the correlation with the parafoveal and perifoveal regions were statistically significant (all *P* < 0.05). For most outer retinal thickness, as the axial length of the eye increased and the diopter decreased, the progression degree of its thickness increased, but the correlation was not statistically significant (see Table 6 for details).

## 4. Discussion

This study revealed that among myopic children, the foveal thickness of the inner retina was significantly thicker in the high myopia group compared to that in the nonhigh myopia group, whereas the parafoveal retina and perifoveal retina displayed a thinner pattern in the high myopia group. However, when considering the full-thickness retina, the fovea was thicker in the high myopia group. When comparing male and female participants, for the inner retina, outer retina, and full retina, the thickness of the foveal and parafoveal region were thicker in males. This pioneering study delved into the relationship between the progression of myopia and variations in retinal thickness among myopic children over a one-year follow-up period. It revealed a significant yet moderate augmentation in retinal thickness across all myopic children, particularly notable in the foveal region compared to the perifoveal region. Intriguingly, the study established a correlation between AL progression and diopter progression with alterations in both the inner retina and the full retina. Furthermore, the thinning of the inner and full retina accelerated with myopia progression.

The previous study on healthy eyes showed that the retinal thickness was not associated with the spherical equivalent refraction (SER), age, or AL [12]. Jin et al. measured the retinal thickness of 7–13 -year-old children and found that the mean central foveal retinal thickness was 234 ± 22 *μ*m in myopic participants and the retinal thickness was thinner in the superior parafoveal and perifoveal regions and in the inferior perifoveal region. In addition, in the study by Jin et al., SER and AL were not correlated with subfoveal/parafoveal retinal thickness, while subfoveal retinal thickness was negatively related to age and positively related to intraocular pressure [8]. Chen et al. reported that central macular thickness was 240.93 ± 17.35 *μ*m and 244.13 ± 22.26 *μ*m in the 6–17 -years-old Chinese low myopia (3.00 *D* < SER 0.50D) and moderate-to-high myopia (SER: 3.00 D) children. The foveal thickness was significantly greater in highly myopic eyes compared with the nonmyopic eyes, while the difference was not statistically significant for low, moderate, and low-to-moderate myopic eyes [13]. In the current study, the foveal thickness of myopic children was 228.02 ± 23.00 *μ*m, which was thinner than the findings of other studies and may be related to the younger age and lower diopter (−1.83 ± 1.29D) in the current study. In children with high myopia, the retina was significantly thicker than that in children with nonhigh myopia. However, during the one-year follow-up, the progression of foveal thickness was not significantly associated with the progression of diopter and AL.

The changes in different regions of the retina differ when the degree of myopia is different. The study by Sam et al. on the thickness of the peripheral retina showed significantly thinned retina in myopic eyes, while the foveal retina and other retinal regions were thicker in myopic eyes. Meanwhile, the peripheral retina was 7% thinner from the nasal region to the temporal region in myopic eyes [14]. Another study found that average parafoveal thickness was thinner in highly myopic eyes without significant difference across all regions [13]. Compared with nonmyopic eyes, the thickness of the superior perifoveal region, inferior perifoveal region, and the temporal perifoveal region were significantly thinner in low, moderate, and highly myopic eyes. In addition, the nasal perifovea was significantly thinned in highly myopic eyes, while the finding for the perifoveal thickness in low-to-moderate myopic eyes was not available due to the lack of adequate data [15, 16]. The current study compared high myopia groups and nonhigh myopia groups found that the full parafoveal and perifoveal retina were relatively thin in children with high myopia in different quadrants, without statistical difference between the two groups. Vurgese et al. reported that AL increase was associated with thinning of the parafoveal retina and was not related to foveal retinal thickness, which was consistent with the hypothesis that axial increase of the globe walls in the midperiphery could be the main driver of myopic globe enlargement [17]. During the 1-year follow-up in the current study, the thickness of the parafoveal and peripheral retina also decreased with the progression of myopia.

The changes in the inner and outer layers of the retina differ in eyes with different degrees of myopia. A study on highly myopic adults revealed thinned outer retina with a noticeably thinned myoid and ellipsoid zone [18]. The inner and outer macula was significantly thinned in the high myopia group. The thinning of the inner and outer retina may be attributable to the myopic globe expansion, with which the inner retina would be tangentially stretched and the anteroposterior tractional force would be imposed on the outer retina [19]. In highly myopic eyes, the outer segment of the receptors layer thickened in the central region, and the increase was associated with the AL growth. Apart from the ganglion cell and inner plexiform layer, the thickness of all layers in the pericentral and peripheral regions changed in highly myopic eyes. Since the inner nuclear layer, combined Henle fiber and outer nuclear layer, and outer segment of receptors layer were all thinner in myopic eyes, the total thickness of the peripheral region was also smaller. However, increased thickness was found in the combined myoid and ellipsoid zone, the combined interdigitation zone, and the retinal pigment epithelium/Bruch complex in myopic eyes [15]. The current study found thickened inner and outer foveal retina in children with high myopia, while the parafoveal and perifoveal inner retina was thinned and the outer retina was thickened, with statistical significance for most of the parameters. During follow-up, with the progression of myopia, the thinning of inner and full parafoveal and perifoveal retina thickness accelerated with a statistical difference. No statistically significant changing trend was found for the outer retina.

There were also reports on the influence of gender on retinal thickness. The central fovea and nasal fovea were found to be thicker in males, urban residents, and those with thinner lens, thinner subfoveal choroid, and longer AL [20]. In terms of the macular thickness across ETDTS regions in males and females, the macular thickness in the foveal, superior inner, nasal inner, inferior inner, temporal inner, and temporal outer quadrants were significantly thicker in males, without significant differences in average macular thickness and average macular volume [21]. Zhang et al. reported that the values of minimum foveal thickness, foveal volume, and average inner ring and temporal outer quadrant macular thickness were significantly greater in males, and no significant difference was found in total macular volume [22]. Macular measurements by gender showed thicker central fovea, average inner macula, and temporal and nasal quadrant outer macula in males [23]. Nevertheless, there was another study revealing a lack of significant association between the central foveal retinal thickness and gender [8]. However, in the current study, the foveal and parafoveal thickness were thinker in male participants with statistical significance, and the perifoveal retina was thinner in male participants with statistical significance.

The relatively short follow-up period, one year, is one of the limitations of the current study. While one-year follow-up revealed the trend of the changes in the thickness of different retinal regions, the trend would be clearer and more definitive with a longer follow-up period. Due to the fact that the participants in this study were mainly 10-year-old schoolchildren with myopia, among whom the number of participants with high myopia was indeed relatively small, the data between the two groups were not well matched. However, we described the data for the high myopia group and the nonhigh myopia group using medians values and performed Wilcoxon rank-sum test to analyze the differences between the two groups, aiming to reduce the impact of data bias. In addition, with the further progression of myopia, in addition to the changes in retinal thickness, the thickness of the choroid would also change significantly, which should be further studied in future research.

Analyses of the diopter, AL, and retinal thickness in school-age myopic children showed that the inner foveal retinal thickness were thicker in highly myopic students than in the nonhighly myopic students, while the parafoveal and perifoveal retina were thinner in highly myopic students. The inner and full retinal thicknesses of male students were thicker than that of females. The progression of myopia mainly affected the changes of the inner retinal thickness in the one-year follow-up.

## Figures and Tables

**Table 1:** Thickness of different retinal regions in 2016.

Variable	Value
Inner fovea (*μ*m)	63.79 ± 10.88
Inner parafovea (*μ*m)	122.02 ± 11.30
Inner paratempo (*μ*m)	118.90 ± 10.98
Inner parasuperior (*μ*m)	124.81 ± 12.76
Inner paranasal (*μ*m)	121.90 ± 11.69
Inner parainferior (*μ*m)	122.39 ± 12.60
Inner perifovea (*μ*m)	103.80 ± 6.90
Inner peritempo (*μ*m)	103.00 ± 7.83
Inner perisuperior (*μ*m)	103.29 ± 7.63
Inner perinasal (*μ*m)	106.55 ± 7.79
Inner periinferior (*μ*m)	102.03 ± 8.38
Outer fovea (*μ*m)	164.23 ± 14.13
Outer parafovea (*μ*m)	188.33 ± 10.31
Outer paratempo (*μ*m)	187.12 ± 10.45
Outer parasuperior (*μ*m)	189.13 ± 13.54
Outer paranasal (*μ*m)	187.83 ± 10.44
Outer parainferior (*μ*m)	188.92 ± 12.31
Outer perifovea (*μ*m)	175.55 ± 9.18
Outer peritempo (*μ*m)	176.55 ± 10.11
Outer perisuperior (*μ*m)	174.32 ± 10.55
Outer perinasal (*μ*m)	178.27 ± 10.57
Outer periinferior (*μ*m)	172.98 ± 11.05
Full fovea (*μ*m)	228.02 ± 23.00
Full parafovea (*μ*m)	310.35 ± 13.22
Full paratempo (*μ*m)	306.03 ± 14.06
Full parasuperior (*μ*m)	313.94 ± 14.77
Full paranasal (*μ*m)	309.70 ± 14.36
Full parainferior (*μ*m)	311.31 ± 14.78
Full perifovea (*μ*m)	279.26 ± 12.96
Full peritempo (*μ*m)	279.55 ± 14.29
Full perisuperior (*μ*m)	277.62 ± 14.53
Full perinasal (*μ*m)	284.82 ± 14.25
Full periinferior (*μ*m)	275.01 ± 16.28

**Table 2:** Comparison of baseline retinal thickness in two myopia subgroups.

Variable	≤−6.00D	>−6.00D	*p*
Inner fovea (*μ*m)	67 [64; 74]	63 [56; 70]	0.033
Inner parafovea (*μ*m)	106 [100; 123]	124 [117; 130]	0.002
Inner paratempo (*μ*m)	108 [101; 116]	120 [114; 126]	0.001
Inner parasuperior (*μ*m)	105 [98; 125]	127 [119; 133]	0.001
Inner paranasal (*μ*m)	109 [102; 126]	123 [117; 129]	0.015
Inner parainferior (*μ*m)	105 [98; 124]	124 [117; 131]	0.004
Inner perifovea (*μ*m)	95.0 [93; 102]	104 [100; 108]	0.001
Inner peritempo (*μ*m)	95.0 [89; 101]	104 [99; 108]	0.001
Inner perisuperior (*μ*m)	94.5 [90; 100]	104 [99; 108]	<0.001
Inner perinasal (*μ*m)	96.5 [94; 104]	107 [102; 112]	0.001
Inner periinferior (*μ*m)	96.5 [94; 102]	103 [97; 108]	0.021
Outer fovea (*μ*m)	174 [171; 178]	166 [158; 173]	0.014
Outer parafovea (*μ*m)	194 [186; 202]	187 [182; 194]	0.037
Outer paratempo (*μ*m)	192 [182; 200]	186 [181; 193]	0.125
Outer parasuperior (*μ*m)	194 [186; 209]	187 [181; 195]	0.027
Outer paranasal (*μ*m)	192 [182; 203]	186 [181; 193]	0.098
Outer parainferior (*μ*m)	194 [188; 203]	187 [181; 196]	0.025
Outer perifovea (*μ*m)	181 [173; 186]	175 [170; 181]	0.059
Outer peritempo (*μ*m)	185 [173; 192]	176 [170; 183]	0.017
Outer perisuperior (*μ*m)	176 [168; 186]	173 [167; 180]	0.470
Outer perinasal (*μ*m)	185 [174; 197]	178 [172; 184]	0.017
Outer periinferior (*μ*m)	174 [171; 182]	172 [167; 179]	0.506
Full fovea (*μ*m)	248 [238; 251]	229 [214; 243]	0.010
Full parafovea (*μ*m)	303 [297; 312]	310 [302; 319]	0.133
Full paratempo (*μ*m)	300 [290; 308]	306 [297; 315]	0.104
Full parasuperior (*μ*m)	305 [295; 319]	314 [305; 323]	0.093
Full paranasal (*μ*m)	310 [300; 319]	309 [301; 319]	0.668
Full parainferior (*μ*m)	304 [300; 311]	312 [303; 320]	0.075
Full perifovea (*μ*m)	271 [268; 290]	279 [271; 288]	0.513
Full peritempo (*μ*m)	278 [270; 290]	280 [271; 289]	0.949
Full perisuperior (*μ*m)	269 [256; 289]	278 [268; 287]	0.233
Full perinasal (*μ*m)	282 [276; 300]	285 [277; 294]	0.822
Full periinferior (*μ*m)	272 [263; 278]	276 [266; 286]	0.219

**Table 3:** Comparison of baseline retinal thickness in two AL subgroups.

Variable	≥26 mm	<26 mm	*P*
Inner fovea (*μ*m)	70 [66; 74]	63 [56; 70]	0.004
Inner parafovea (*μ*m)	105 [102; 118]	124 [117; 130]	<0.001
Inner paratempo (*μ*m)	107 [102; 114]	120 [114; 126]	0.001
Inner parasuperior (*μ*m)	106 [102; 120]	127 [119; 133]	<0.001
Inner paranasal (*μ*m)	110 [103; 114]	123 [117; 129]	<0.001
Inner parainferior (*μ*m)	108 [100; 117]	124 [117; 131]	0.001
Inner perifovea (*μ*m)	94 [91; 98]	104 [100; 108]	<0.001
Inner peritempo (*μ*m)	94 [88; 98]	104 [99; 108]	<0.001
Inner perisuperior (*μ*m)	98 [91; 103]	104 [99; 108]	0.006
Inner perinasal (*μ*m)	96 [90; 100]	107 [102; 112]	<0.001
Inner periinferior (*μ*m)	97 [88; 99]	103 [97; 108]	<0.001
Outer fovea (*μ*m)	175 [169; 182]	166 [158; 173]	0.013
Outer parafovea (*μ*m)	196 [190; 205]	187 [181; 194]	0.001
Outer paratempo (*μ*m)	194 [187; 203]	186 [181; 193]	0.007
Outer parasuperior (*μ*m)	199 [186; 216]	187 [181; 195]	0.008
Outer paranasal (*μ*m)	196 [190; 205]	186 [181; 193]	0.001
Outer parainferior (*μ*m)	199 [187; 205]	187 [181; 195]	0.011
Outer perifovea (*μ*m)	174 [172; 186]	175 [170; 181]	0.261
Outer peritempo (*μ*m)	176 [172; 190]	176 [170; 183]	0.276
Outer perisuperior (*μ*m)	174 [165; 184]	174 [167; 180]	0.803
Outer perinasal (*μ*m)	182 [174; 192]	178 [172; 184]	0.079
Outer periinferior (*μ*m)	173 [167; 182]	172 [167; 179]	0.730
Full fovea (*μ*m)	246 [238; 252]	229 [214; 243]	0.004
Full parafovea (*μ*m)	310 [299; 317]	310 [302; 319]	0.501
Full paratempo (*μ*m)	300 [296; 315]	306 [297; 315]	0.512
Full parasuperior (*μ*m)	317 [302; 324]	314 [305; 323]	0.549
Full paranasal (*μ*m)	306 [300; 322]	310 [301; 319]	0.711
Full parainferior (*μ*m)	305 [302; 316]	312 [303; 320]	0.218
Full perifovea (*μ*m)	271 [268; 285]	279 [271; 288]	0.085
Full peritempo (*μ*m)	273 [262; 289]	280 [271; 289]	0.178
Full perisuperior (*μ*m)	272 [258; 289]	278 [268; 287]	0.300
Full perinasal (*μ*m)	280 [272; 294]	285 [277; 295]	0.304
Full periinferior (*μ*m)	270 [262; 272]	276 [266; 286]	0.017

**Table 4:** Comparison of baseline retinal thickness in male and female participants.

Variable	Male	Female	*P*
Inner fovea (*μ*m)	65.33 ± 10.91	62.34 ± 10.65	<0.001
Inner parafovea (*μ*m)	122.91 ± 11.56	121.19 ± 10.98	0.010
Inner paratempo (*μ*m)	119.68 ± 11.35	118.17 ± 10.58	0.019
Inner parasuperior (*μ*m)	125.78 ± 13.21	123.89 ± 12.27	0.012
Inner paranasal (*μ*m)	122.74 ± 12.00	121.11 ± 11.35	0.018
Inner parainferior (*μ*m)	123.34 ± 12.91	121.50 ± 12.24	0.013
Inner perifovea (*μ*m)	103.58 ± 6.85	104.01 ± 6.94	0.294
Inner peritempo (*μ*m)	102.97 ± 7.83	103.03 ± 7.83	0.890
Inner perisuperior (*μ*m)	103.11 ± 7.44	103.46 ± 7.81	0.430
Inner perinasal (*μ*m)	106.57 ± 7.58	106.54 ± 7.99	0.954
Inner periinferior (*μ*m)	101.57 ± 8.20	102.46 ± 8.52	0.072
Outer fovea (*μ*m)	166.59 ± 12.96	162.01 ± 14.82	<0.001
Outer parafovea (*μ*m)	189.41 ± 10.86	187.31 ± 9.66	0.001
Outer paratempo (*μ*m)	188.33 ± 11.15	185.99 ± 9.63	<0.001
Outer parasuperior (*μ*m)	190.21 ± 13.79	188.11 ± 13.23	0.008
Outer paranasal (*μ*m)	188.90 ± 11.06	186.81 ± 9.72	0.001
Outer parainferior (*μ*m)	189.99 ± 12.64	187.90 ± 11.91	0.004
Outer perifovea (*μ*m)	175.86 ± 9.16	175.26 ± 9.19	0.264
Outer peritempo (*μ*m)	177.27 ± 10.08	175.87 ± 10.11	0.019
Outer perisuperior (*μ*m)	174.42 ± 9.82	174.23 ± 11.20	0.753
Outer perinasal (*μ*m)	178.79 ± 10.40	177.78 ± 10.71	0.104
Outer periinferior (*μ*m)	172.96 ± 11.04	173.00 ± 11.08	0.956
Full fovea (*μ*m)	231.91 ± 21.72	224.35 ± 23.59	<0.001
Full parafovea (*μ*m)	312.31 ± 13.13	308.50 ± 13.04	<0.001
Full paratempo (*μ*m)	308.01 ± 13.95	304.16 ± 13.92	<0.001
Full parasuperior (*μ*m)	315.99 ± 14.41	312.00 ± 14.85	<0.001
Full paranasal (*μ*m)	311.57 ± 14.46	307.92 ± 14.05	<0.001
Full parainferior (*μ*m)	313.33 ± 14.58	309.40 ± 14.73	<0.001
Full perifovea (*μ*m)	279.37 ± 12.56	279.16 ± 13.34	0.786
Full peritempo (*μ*m)	280.24 ± 13.84	278.90 ± 14.68	0.112
Full perisuperior (*μ*m)	277.53 ± 13.67	277.69 ± 15.31	0.851
Full perinasal (*μ*m)	285.36 ± 13.40	284.32 ± 15.00	0.215
Full periinferior (*μ*m)	274.54 ± 15.86	275.46 ± 16.67	0.336

**Table 5:** Progression in AL, diopter, and retinal thickness.

Variable	Progression
Diopter (*d*)	−0.27 ± 1.09
Axial length (mm)	0.33 ± 0.39
Inner fovea (*μ*m)	1.77 ± 9.95
Inner parafovea (*μ*m)	1.90 ± 13.81
Inner paratempo (*μ*m)	1.48 ± 12.79
Inner parasuperior (*μ*m)	2.14 ± 16.24
Inner paranasal (*μ*m)	1.06 ± 13.14
Inner parainferior (*μ*m)	3.01 ± 16.01
Inner perifovea (*μ*m)	0.46 ± 7.23
Inner peritempo (*μ*m)	0.92 ± 8.53
Inner perisuperior (*μ*m)	0.38 ± 7.57
Inner perinasal (*μ*m)	0.30 ± 8.41
Inner periinferior (*μ*m)	0.52 ± 8.44
Outer fovea (*μ*m)	3.73 ± 12.75
Outer parafovea (*μ*m)	0.36 ± 11.45
Outer paratempo (*μ*m)	1.04 ± 10.67
Outer parasuperior (*μ*m)	−0.25 ± 16.23
Outer paranasal (*μ*m)	1.29 ± 11.63
Outer parainferior (*μ*m)	−0.52 ± 14.57
Outer perifovea (*μ*m)	1.23 ± 8.80
Outer peritempo (*μ*m)	1.43 ± 9.94
Outer perisuperior (*μ*m)	0.74 ± 9.94
Outer perinasal (*μ*m)	1.70 ± 11.26
Outer periinferior (*μ*m)	1.03 ± 11.47
Full fovea (*μ*m)	5.50 ± 19.41
Full parafovea (*μ*m)	2.26 ± 8.83
Full paratempo (*μ*m)	2.53 ± 10.39
Full parasuperior (*μ*m)	1.80 ± 12.78
Full paranasal (*μ*m)	2.31 ± 11.45
Full parainferior (*μ*m)	2.49 ± 12.24
Full perifovea (*μ*m)	1.69 ± 9.71
Full peritempo (*μ*m)	2.34 ± 11.62
Full perisuperior (*μ*m)	1.11 ± 10.01
Full perinasal (*μ*m)	2.01 ± 11.96
Full periinferior (*μ*m)	1.55 ± 13.76

**Table 6:** Correlation of the progression in AL and diopter with the progression in retinal thickness.

	Diopter		Axial length	
	Spearman's rank correlation coefficient	*P*	Spearman's rank correlation coefficient	*P*
Inner fovea (*μ*m)	0.023	0.439	−0.002	0.956
Inner parafovea (*μ*m)	0.079	0.007	−0.102	0.001
Inner paratempo (*μ*m)	0.059	0.045	−0.097	0.001
Inner parasuperior (*μ*m)	0.070	0.017	−0.111	<0.001
Inner paranasal (*μ*m)	0.072	0.014	−0.071	0.016
Inner parainferior (*μ*m)	0.087	<0.001	−0.084	0.005
Inner perifovea (*μ*m)	0.138	<0.001	−0.165	<0.001
Inner peritempo (*μ*m)	0.118	<0.001	−0.117	<0.001
Inner perisuperior (*μ*m)	0.103	<0.001	−0.163	<0.001
Inner perinasal (*μ*m)	0.120	<0.001	−0.153	<0.001
Inner periinferior (*μ*m)	0.139	<0.001	−0.145	<0.001
Outer fovea (*μ*m)	0.004	0.883	0.010	0.729
Outer parafovea (*μ*m)	−0.020	0.500	0.019	0.517
Outer paratempo (*μ*m)	−0.005	0.855	0.018	0.535
Outer parasuperior (*μ*m)	−0.021	0.475	0.032	0.276
Outer paranasal (*μ*m)	0.012	0.687	−0.024	0.421
Outer parainferior (*μ*m)	−0.034	0.243	0.011	0.712
Outer perifovea (*μ*m)	−0.050	0.087	0.004	0.888
Outer peritempo (*μ*m)	−0.013	0.663	−0.016	0.580
Outer perisuperior (*μ*m)	−0.036	0.225	0.018	0.539
Outer perinasal (*μ*m)	−0.054	0.064	−0.002	0.942
Outer periinferior (*μ*m)	−0.035	0.237	−0.013	0.657
Full fovea (*μ*m)	0.009	0.759	0.006	0.838
Full parafovea (*μ*m)	0.086	0.003	−0.137	<0.001
Full paratempo (*μ*m)	0.070	0.017	−0.107	<0.001
Full parasuperior (*μ*m)	0.082	0.005	−0.125	<0.001
Full paranasal (*μ*m)	0.077	0.009	−0.107	<0.001
Full parainferior (*μ*m)	0.096	0.001	−0.137	<0.001
Full perifovea (*μ*m)	0.082	0.005	−0.156	<0.001
Full peritempo (*μ*m)	0.086	0.004	−0.105	<0.001
Full perisuperior (*μ*m)	0.051	0.082	−0.143	<0.001
Full perinasal (*μ*m)	0.077	0.009	−0.148	<0.001
Full periinferior (*μ*m)	0.073	0.013	−0.140	<0.001

## Data Availability

All data generated or analyzed during this study are included in this article. Further enquiries can be directed to the corresponding author.
